# Incidence, socioeconomic deprivation, volume-outcome and survival in adult patients with acute lymphoblastic leukaemia in England

**DOI:** 10.1186/s12885-017-3975-0

**Published:** 2018-01-04

**Authors:** Ravi Maheswaran, Nick Morley

**Affiliations:** 1 Public Health GIS Unit, School of Health and Related Research, University of Sheffield, Regent Court, 30 Regent Street, Sheffield, S1 4DA UK; 2 Department of Haematology, Royal Hallamshire Hospital, Glossop Road, Sheffield, S10 2JF UK

**Keywords:** Acute lymphoblastic leukaemia, Incidence, Survival, Mortality, Socioeconomic deprivation

## Abstract

**Background:**

We examined incidence and survival in relation to age, gender, socioeconomic deprivation, rurality and trends over time. We also examined the association between volume of patients treated by hospitals and survival.

**Methods:**

Incident cases (2001–12) were identified using comprehensive National Health Service admissions data for England, with follow-up to March 2013. Socioeconomic deprivation was based on census area of residence. Volume was assessed in a three-year subset of the data with consistent hospital provider codes.

**Results:**

There were 2921 adults aged 18 or more years diagnosed with acute lymphoblastic leukaemia (ALL) in the 12-year time span, giving a crude annual incidence of 0.61/100,000 population. Five-year survival was 32% (1870 deaths).

Compared with patients living in least deprived areas, survival was worse for patients living in intermediate and most deprived areas, with mortality hazard ratios 21% (95% CI 8–35%) and 16% (95% CI 3–30%) higher respectively.

Hospitals treating low volumes of adults with ALL were associated with poorer survival. The adjusted mortality hazard ratio in this subset of 465 patients was 33% (95% CI 3–73%) higher in low volume hospitals.

There was no evidence of association between socioeconomic deprivation and incidence. Rurality did not appear to be associated with incidence or survival. Incidence was higher in men but there was no evidence of a gender difference in survival. Survival improved over time.

**Conclusion:**

The associations between socioeconomic deprivation and survival and between volume and outcome for adults with ALL, if confirmed, are likely to have significant implications for the organisation of services for adults with ALL.

## Background

Acute lymphoblastic leukaemia (ALL) is a haematological malignancy arising from lymphoblasts. Whilst it is a relatively common malignancy in children with a higher incidence in boys, the incidence is rare in adults. The prognosis is good in childhood ALL but is poor in the adult form of the disease [1]. The epidemiology of childhood ALL has been well documented but much less has been done on the epidemiology in adults.

Socioeconomic inequalities in health are a major cause of concern and socioeconomic deprivation is associated with the incidence and prognosis of many conditions [2]. With regard to the incidence of ALL in childhood, a review of early studies found that higher incidence appeared to be associated with higher socioeconomic status [3]. However, another review which included several subsequent studies found conflicting evidence [4]. Little has been done on the incidence of ALL in adults in relation to socioeconomic deprivation and a study of young adults found no evidence of association [5].

The association between socioeconomic deprivation and survival has been examined in a number of studies on childhood ALL, with poorer survival associated with deprivation in several studies but not others [5–10]. Little research, however, has been carried out on the association between socioeconomic deprivation and survival in adults with ALL, with no significant association found in young adults [5].

The association between the volume of patients treated at a hospital and outcome has significant implications for the organisation and centralisation of services and has been investigated in relation to an increasing range of conditions, including medical and surgical care for cancer patients [11]. With regard to ALL, centres treating a high volume of childhood ALL cases were found to have better outcomes in some studies but not others [12–14]. However, the association between volume and outcome has not been investigated in relation to ALL in adults.

Trends over time and geographical variation are other aspects relevant to the epidemiology of ALL in adults. In one region in the UK, there appeared to be no improvement in survival of young adults diagnosed between 1990 and 2002 [5]. However, studies in other countries which included more recent years have reported improvements in survival over time [15, 16]. International and within country geographical variation in ALL incidence and survival has been examined, although mainly in children or with all ages combined [1, 5, 10]. Little, however, has been done on the differences in incidence and survival between urban and rural areas in relation to adults with ALL.

We examined the incidence and survival of adults with ALL in relation to age, gender, socioeconomic deprivation, rurality and trends over time. We also examined the association between volume of patients treated by hospital providers and survival.

## Methods

### Study design, area and time span

We used a population (ecological) study design to investigate incidence and a cohort study design to examine survival amongst all adult patients (aged 18 years or more at diagnosis) in England diagnosed from 2001 to 2012. Follow-up was to 31st March 2013. The study was approved by the University of Sheffield’s Research Ethics Committee. Participant consent was not necessary as this study involved the use of de-identified Hospital Episode Statistics (HES) supplied by NHS Digital. NHS Digital is an executive non-departmental public body accountable to Parliament and its statutory role is set out in the Health and Social Care Act 2012.

### Data on cases

We used pseudoanonymised HES data with linked mortality data to identify cases with ALL. HES is a data warehouse containing details of all admissions to National Health Service (NHS) hospitals in England [17]. Admissions are recorded as “episodes” where an episode is a period of care under a single consultant during an admission. An admission may comprise more than one episode if a patient is transferred from the care of one consultant to another during the admission, though in practice most admissions comprise a single episode. The admission episode record includes several diagnosis and procedure code fields. We used the first seven diagnosis fields and the first 12 procedure code fields in order to use a set that was consistent throughout the study time span (there were more fields in later years). HES years follow the UK financial calendar and run from 1st April to 31st March the following year. We obtained data from 1st April 2000 to 31st March 2013. We excluded patients with a first admission with ALL in 2000 to exclude prevalent cases.

We used the International Classification of Diseases, 10th revision (ICD-10) code C91.0 to identify cases of ALL. The main advantage of using HES is complete national data capture as every adult in England who developed ALL will almost certainly have been admitted to an NHS hospital on at least one occasion for confirmation of the diagnosis and assessment for treatment and supportive care. However, there is significant scope for error in coding of leukaemia in HES.

We therefore first identified individuals (using the HES pseudoanonymised identifier) with a C91.0 code in any diagnosis field and extracted all their admission episode records. We then carried out a whole series of exploratory analyses which included examining procedure codes for chemotherapy, central line insertion, spinal fluid investigation and bone marrow examination. We also examined numbers of admissions per patient, cause of death and diagnoses likely to be miscoded as ALL. We classified the latter as “ambiguous” diagnoses, which included other leukaemias (C91.1 – C96.9) and lymphomas and myeloma (C81.0 – C90.2).

We arrived at a definition of a case as a patient with all of the following: a diagnosis of C91.0 as the primary diagnosis in at least one episode, an admission count >1; a chemotherapy code recorded in at least one episode; and a ratio of count of episodes with C91.0 code to count of episodes containing an ambiguous diagnosis code >1. Patients admitted for assessment but deemed too frail to withstand chemotherapy, or who died during or following their first admission, would have been excluded by the above definition. We therefore also included, as a separate definition, patients with C91.0 as the cause of death. These patients would also have had a C91.0 code in any diagnosis field in at least one admission episode. Records, in at least one episode, of central line insertion (65.6%), spinal fluid investigation (54.1%) or bone marrow examination (73.7%) were too incomplete to be used in the case definition, given that all patients undergoing diagnosis and treatment would have had these procedures carried out. The average number of cases identified using the above criteria was 243 per year and was comparable to the average estimated count of 240 per year from Office for National Statistics (ONS) statistics based on cancer registries [18].

### Other data

We used the Income Domain of the Index of Multiple Deprivation (IMD) 2007 as an indicator of socioeconomic deprivation at the small area level [19]. The IMD is a standard deprivation indicator used by local and national government and is available at the lower layer super-output area (LSOA) level. LSOAs are census areas used in the 2001 and 2011 censuses, with approximately 1500 people per LSOA.

We used the government’s urban-rural classification to classify LSOAs as either urban or rural [20]. Mid-year population counts by five-year age band and sex at the LSOA level were also available from ONS.

### Volume and outcome

HES data contain provider unit codes which we used to estimate the volume of adult patients with ALL treated at each provider hospital. Provider units have reconfigured over time on several occasions with consequent changes in codes. We had a consistent set of codes for a limited time span (1st April 2008 – 31st March 2011) available from another project, which we used for this element of the analysis [21]. Estimation of volume was based on new adult patients with ALL diagnosed during this three-year time span. Patients could have been admitted to more than one hospital during this time span and were included in the volume count of each hospital they were admitted to. We used the median to split hospitals into high and low volume hospitals.

### Statistical analysis

We used Poisson regression to examine incidence, and survival plots and Cox regression to examine survival. Age (20–29 years for incidence, 18–29 years for survival, then 10-year bands to 80+ years), gender, rurality, time-period (3 four-year periods) and socioeconomic deprivation (categorised by tertile) were entered as categorical variables. The logs of population counts were entered as the offset in the Poisson regression, which was restricted to 31,672 (97.5%) of the 32,482 LSOAs in 2001 which remained unchanged in 2011. Log [−log] plots were consistent with the proportionality assumption for Cox regression. There was no overdispersion in the Poisson model.

Date of diagnosis and age at diagnosis were based on the admission date of the first admission with a C91.0 code in any diagnosis field. Survival time was calculated in years from the date of diagnosis to date of death or censored at the end of the follow-up period. Analysis of the effect of volume on survival was examined on the subset of new patients described above. Results are presented as rate ratios or hazard ratios, adjusted for all other variables, with 95% confidence intervals.

## Results

### Characteristics of patients

There were a total of 2921 adults aged 18 or more years diagnosed with ALL in the 12-year time span examined, giving a crude annual incidence of 0.61/100,000 population. There were 1870 deaths in 7078 person-years of follow-up time.

The characteristics of the patients are shown in Table 1. Almost a quarter of the patients were in the 18–29 year age group, 42.5% were women and 18.6% lived in rural areas. There was a median of 23 (Interquartile Range (IQR) 6–53) admissions per patient within the time period examined. A very high percentage of admission episodes for each patient had a diagnosis of ALL recorded in one of the diagnosis fields (median 95% (IQR 81–100%)), with only a very small percentage of episodes containing an ambiguous diagnosis code (median 0% (IQR 0–5%).

**Table 1:** Characteristics of adult patients with ALL in England, 2001–12

Characteristic	N (%)
Age (y)	
- 18-29	710 (24.3)
- 30-39	372 (12.7)
- 40-49	401 (13.7)
- 50-59	426 (14.6)
- 60-69	469 (16.1)
- 70-79	361 (12.4)
- 80+	182 (6.2)
Women	1241 (42.5)
Socioeconomic deprivation (by tertile)	
- Least deprived	974 (33.3)
- Intermediate	974 (33.3)
- Most deprived	973 (33.3)
Living in rural areas	544 (18.6)
Time period	
- 2001-04	916 (31.4)
- 2005-08	1011 (34.6)
- 2009-12	994 (34.0)
Deaths	1870 (64.0)
Total patients	2921
	Median (IQR)
Number of admissions per patient	23 (6–53)
Percentage of admission episodes per patient with C91.0 in any diagnosis field	95 (81–100)
Percentage of admission episodes per patient with an ambiguous diagnosis in any diagnosis field	0 (0–5)
Ratio per patient of admission episodes with an ambiguous diagnosis to episodes with C91.0	0 (0–0.1)
Percentage of admission episodes per patient with a chemotherapy code	35 (17–56)

### Incidence rate ratios

The adjusted incidence rate ratios are shown in Table 2. The incidence rate ratio was 42% (31–53%) higher in men. Compared with the 20–29 year age group, incidence was lower in patients aged 30–59 years and higher in patients aged 60+ years. There was no evidence of association of incidence with socioeconomic deprivation or rurality and no evidence of variation over the time frame examined.

**Table 2:** Adjusted iIncidence rate ratios (95% CI) for ALL in adults in England, 2001–12

Characteristic	Adjusted rate ratio (95% CI)
Age (y)	
- 20-29	1
- 30-39	0.65 (0.57–0.74)
- 40-49	0.70 (0.62–0.81)
- 50-59	0.87 (0.76–0.99)
- 60-69	1.19 (1.05–1.35)
- 70-79	1.30 (1.13–1.49)
- 80+	1.09 (0.92–1.29)
Gender	
- Women	1
- Men	1.42 (1.31–1.53)
Socioeconomic deprivation (by tertile)	
- Least deprived	1
- Intermediate	0.92 (0.84–1.01)
- Most deprived	0.97 (0.88–1.07)
Urban or rural areas	
- Urban	1
- Rural	0.95 (0.86–1.05)
Time period	
- 2001-04	1
- 2005-08	1.01 (0.92–1.11)
- 2009-12	0.99 (0.90–1.09)

### Survival

**Fig. 1:**
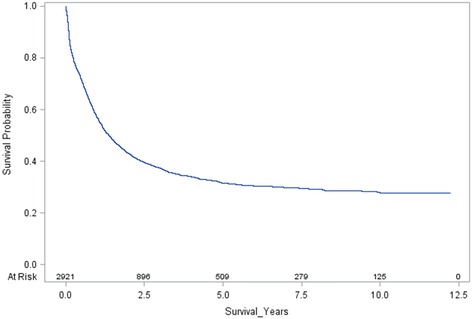
Survival plot for adults with ALL in England, 2001–12

Figure 1 shows the survival plot for adults diagnosed with ALL in England from 2001 to 12. The overall five-year survival for this cohort of 2921 patients was 32%. When restricted to the 2559 patients who had a record of chemotherapy, the five-year survival was 36%. The adjusted hazard ratios for mortality are shown in Table 3. These were based on analysis of all 2921 patients. Hazard ratios increased with increasing age but there was no evidence of a gender difference in survival. Compared with patients living in the least deprived areas, survival was poorer in patients living in intermediate and most deprived areas, with mortality hazard ratios 21% (8–35%) and 16% (3–30%) higher respectively. In terms of five-year survival, predicted rates for the least, intermediate and most deprived areas were 36% (33–38%), 30% (28–33%) and 31% (29–34%) respectively.

**Table 3:** Adjusted hazard ratios (95% CI) for mortality in adults with ALL in England, 2001–12

Characteristic	Adjusted hazard ratio (95% CI) for all 2921 patients
Age (y)	
- 18-29	1
- 30-39	1.38 (1.15–1.66)
- 40-49	1.69 (1.42–2.01)
- 50-59	2.23 (1.89–2.62)
- 60-69	3.72 (3.18–4.34)
- 70-79	5.50 (4.68–6.46)
- 80+	9.02 (7.43–10.95)
Gender	
- Women	1
- Men	1.04 (0.95–1.14)
Socioeconomic deprivation (by tertile)	
- Least deprived	1
- Intermediate	1.21 (1.08–1.35)
- Most deprived	1.16 (1.03–1.30)
Urban or rural areas	
- Urban	1
- Rural	1.00 (0.89–1.13)
Time period	
- 2001-04	1
- 2005-08	0.93 (0.84–1.03)
- 2009-12	0.70 (0.62–0.79)
	Adjusted hazard ratio (95% CI) for subset of 465 patients^a^
Record of chemotherapy	
- Yes	1
- No	5.53 (3.98–7.69)
Hospital provider volume of ALL patients	
- >15	1
- = <15	1.33 (1.03–1.73)

^a^Patients diagnosed between 1st April 2008 and 31st March 2011 with all their admissions at a single hospital provider unit during this period. This analysis included chemotherapy and volume in addition to the other variables

There was no association with rurality. Survival improved over the time period examined, and the mortality hazard ratio was 30% (21–38%) lower in patients presenting in 2009–12 compared with patients presenting in 2001–04. In terms of five-year survival, predicted rates for patients presenting in 2001–04, 2005–08 and 2009–12 were 28% (26–31%), 30% (28–33%) and 39% (36–42%) respectively.

### Volume-outcome association

There were 727 new patients admitted between 1st April 2008 and 31st March 2011, the period for which we had a consistent set of provider unit codes available. Of these, 465 had all their admissions to the same provider unit during this time span. The adjusted hazard ratio for mortality in this subset of 465 patients was 33% (3–73%) higher in low volume hospitals (Table 3). This analysis additionally adjusted for chemotherapy treatment as patients with no record of treatment for chemotherapy had much poorer survival.

## Discussion

Whilst we found no evidence of association between socioeconomic deprivation and incidence of ALL in adults, survival with higher amongst patients living in affluent areas. Hospitals treating low volumes of adults with ALL were associated with poorer survival. Rurality did not appear to be associated with incidence or survival. Incidence was higher in men but there was no evidence of a gender difference in survival. Survival improved over time but was poor amongst older patients.

A review of early studies of childhood ALL found that higher incidence was associated with higher socioeconomic status [3]. However, a subsequent review found that the evidence was mixed, with several studies reporting lower incidence of childhood ALL in more affluent groups [4]. A more recent study found a non-significant decrease in incidence with increasing affluence in analyses combining children and adults [22]. Another study found no evidence of association between incidence and deprivation in young adults, consistent with our results for all adults [5].

With regard to survival, a national study in the UK on childhood ALL found that higher levels of socioeconomic deprivation were associated with poorer survival [9]. The authors argued that the association was not due to different treatment across social groups as the association was also seen in children enrolled in the UKALL XI trial. Our results indicate that socioeconomic inequalities in survival are also a significant cause for concern in adults with ALL and further research is required to understand and address this issue.

Our finding that mortality was higher for adults with ALL treated at hospitals treating low volumes of adult ALL patients is of concern. An early study on volume and outcome in the treatment of childhood ALL found that centres treating an average of six or more patients per year had the best long-term survival rates [12]. Although two recent childhood ALL studies found no association between volume and outcome, they only examined mortality during induction therapy [13, 14]. A study of in-hospital mortality in patients undergoing chemotherapy for acute myeloid leukaemia, a disease predominantly of adults, was the first to report that the mortality rate was significantly higher in low-volume hospitals [23]. High volume centres in general are more likely to have specialised clinical teams and more experienced staff to manage patients with specific medical conditions, which could explain better outcomes [24].

Our finding of improved survival over the time period examined is consistent with studies of adults with ALL in Germany, the USA and the Netherlands [15, 16]. We found no gender differences in survival. Others have reported that whilst survival was worse in men previously, the gender difference appears to no longer be apparent [15]. Survival, however, remains poor, especially amongst older adults and those with relapsed or refractory disease [1, 25].

Our study has a number of potential limitations which need to be considered. We used HES data which has the advantage of being a comprehensive national system and all adults with ALL are likely to have been admitted at least once to confirm the diagnosis. However, coding and data entry errors could have led to over or underascertainment of cases. We examined the data in detail in order to arrive at a set of procedures to keep errors to a minimum. The average case counts we obtained were comparable to the ONS estimates from cancer registration data. In addition, the five-year survival of 36% we observed for patients receiving chemotherapy is consistent with the five-year survival of 38% for adults in the UKALL 12 trial [26]. There may have been errors in the mid-year population estimates we used to calculate incidence rates. We used an area based measure of socioeconomic deprivation assigned to individual patients and some patients may have been misclassified. However, we used a measure based on small geographical areas which would have minimised this error as levels of socioeconomic deprivation for populations in smaller geographical units are likely to be generally more homogenous. The volume indicator was based on a relatively small number of cases because of the limited time span for which we had consistent provider unit codes, and some providers may have been misclassified by volume. However, misclassification in this context might have diluted the strength of the association. Potential errors need to be taken into account in the interpretation of our results.

## Conclusions

In terms of future research and policy, further work is needed to confirm the association between higher socioeconomic deprivation and poorer survival and the observation that hospitals treating a low volume of patients were associated with poorer outcome for adults with ALL. These findings, if confirmed, are likely to have significant implications for the organisation of services for the treatment of adults with ALL.

## Abbreviations

ALL: Acute lymphoblastic leukaemia
HES: Hospital episode statistics
ICD-10: International classification of diseases, 10th revision
IMD: Index of multiple deprivation
IQR: Interquartile range
LSOA: Lower layer super-output area
NHS: National Health Service
ONS: Office for national statistics
